# Microglia cannibalism and efferocytosis leads to shorter lifespans of developmental microglia

**DOI:** 10.1371/journal.pbio.3002819

**Published:** 2024-10-30

**Authors:** Hannah Gordon, Zachary T. Schafer, Cody J. Smith

**Affiliations:** 1 Department of Biological Sciences at the University of Notre Dame, Notre Dame, Indiana, United States of America; 2 The Center for Stem Cells and Regenerative Medicine at the University of Notre Dame, Notre Dame, Indiana, United States of America; UCSD, UNITED STATES OF AMERICA

## Abstract

The overproduction of cells and subsequent production of debris is a universal principle of neurodevelopment. Here, we show an additional feature of the developing nervous system that causes neural debris—promoted by the sacrificial nature of embryonic microglia that irreversibly become phagocytic after clearing other neural debris. Described as long-lived, microglia colonize the embryonic brain and persist into adulthood. Using transgenic zebrafish to investigate the microglia debris during brain construction, we identified that unlike other neural cell types that die in developmental stages after they have expanded, necroptosis-dependent microglial debris is prevalent when microglia are expanding in the zebrafish brain. Time-lapse imaging of microglia demonstrates that this debris is cannibalized by other microglia. To investigate features that promote microglia death and cannibalism, we used time-lapse imaging and fate-mapping strategies to track the lifespan of individual developmental microglia. These approaches revealed that instead of embryonic microglia being long-lived cells that completely digest their phagocytic debris, once most developmental microglia in zebrafish become phagocytic they eventually die, including ones that are cannibalistic. These results establish a paradox—which we tested by increasing neural debris and manipulating phagocytosis—that once most microglia in the embryo become phagocytic, they die, create debris, and then are cannibalized by other microglia, resulting in more phagocytic microglia that are destined to die.

## Abstract 2

The microglia population is considered long lived. This study demonstrates that individual embryonic microglia die within 3 days after clearing neural debris and are cannibalized by existing microglia which paradoxically leads to additional microglial death.

Programmed cell death and clearance of that debris is a universal principle of nervous system development [1,2]. In vertebrates, this debris is cleared by microglia, which are the resident and professional phagocyte of the brain [3]. Microglia are considered long-lived cells that colonize the embryonic brain from the embryonic yolk-sac [3–7]. This definition is supported by fate-mapping studies that demonstrate yolksac derived cells generate microglia that persist into adulthood and intravital imaging of adult mouse brains that demonstrate low turnover of microglia in the healthy animals [5–8]. Genetic-based lineage-tracing methodologies in zebrafish have also demonstrated embryonic rostral blood island-derived microglia are present in the brain until 15 days post fertilization (dpf) [9,10]. Adult zebrafish microglia are also derived from progenitors that can be labeled in the embryo [10–12]. While these genetic experiments clearly demonstrate that microglia in embryos and adults are produced from embryonic sources, they do not label microglia distinct from their progenitors nor do they allow for sparse labeling that can track the fate of individual microglia. Time-lapse imaging of zebrafish microglia has confirmed that the embryonic microglia population is stable over 24-h periods [13–16]. Conflicting the long-lived nature of individual microglia is evidence of microglia turnover in zebrafish and humans during development and of microglia death in adult mice [9,17,18]. Thus while the microglia population is stable and long-lived, the lifespan of individual microglia in the embryonic brain remains a concept that is unanswered.

To understand the lifespan of individual microglia, we searched for microglia death during brain construction. Using zebrafish to precisely fate-map individual microglia, we found that once most embryonic microglia become phagocytic, they die. Unique from other cells in development, this death is necroptotic. The scale of this death requires a rapid turnover of microglia when they are expanding in the brain. Unlike the adult brain states when microglia are tiled [19], our evidence in zebrafish shows that developmental microglia cannibalize microglia debris, resulting in microglia that are destined to die because they are phagocytic. In addition to introducing a new process that produces developmental cell death, this work demonstrates that embryonic microglia lifespan rarely exceeds 3 days when phagocytic debris is prominent.

To explore microglia death in the developing nervous system, we first searched for signatures of microglia death in the developing brain using transgenic zebrafish, *Tg(pu1*:*Gal4-UAS*:*GFP)* (hereafter *pu1*:*GFP*) that use regulatory regions of *pu1* to label microglia populations [15]. To ensure that we could identify death regardless of the mode of death, we first scored the amount of GFP^+^ debris in the brain from 3 to 5 dpf. In these ages, we scored that GFP^+^ microglia debris was present and increased across days (Fig 1A and 1B; *N* = 21 animals, *p* = 0.0397 3 dpf versus 5 dpf, post hoc Tukey test). We confirmed this debris was derived from microglia and not from other *pu1*^*+*^ macrophages that may enter the embryonic brain by demonstrating they also label with the zebrafish microglia-specific antibody 4C4 (Fig 1C) [20,21]. 4C4 does label a subset of macrophages but they are localized outside the brain region in wild-type animals. To complement the 4C4 labeling, we also used time-lapse imaging to visualize death in *Tg(pu1*:*GFP)* animals at 4 dpf and then scored each cells ramified morphology that is typical of microglia. The projection length and number of projections between microglia that were fated to die compared to 4C4^+^ microglia were indistinguishable (Figs 1D–1F and S1A), consistent with their microglia identity.

**Fig 1:**
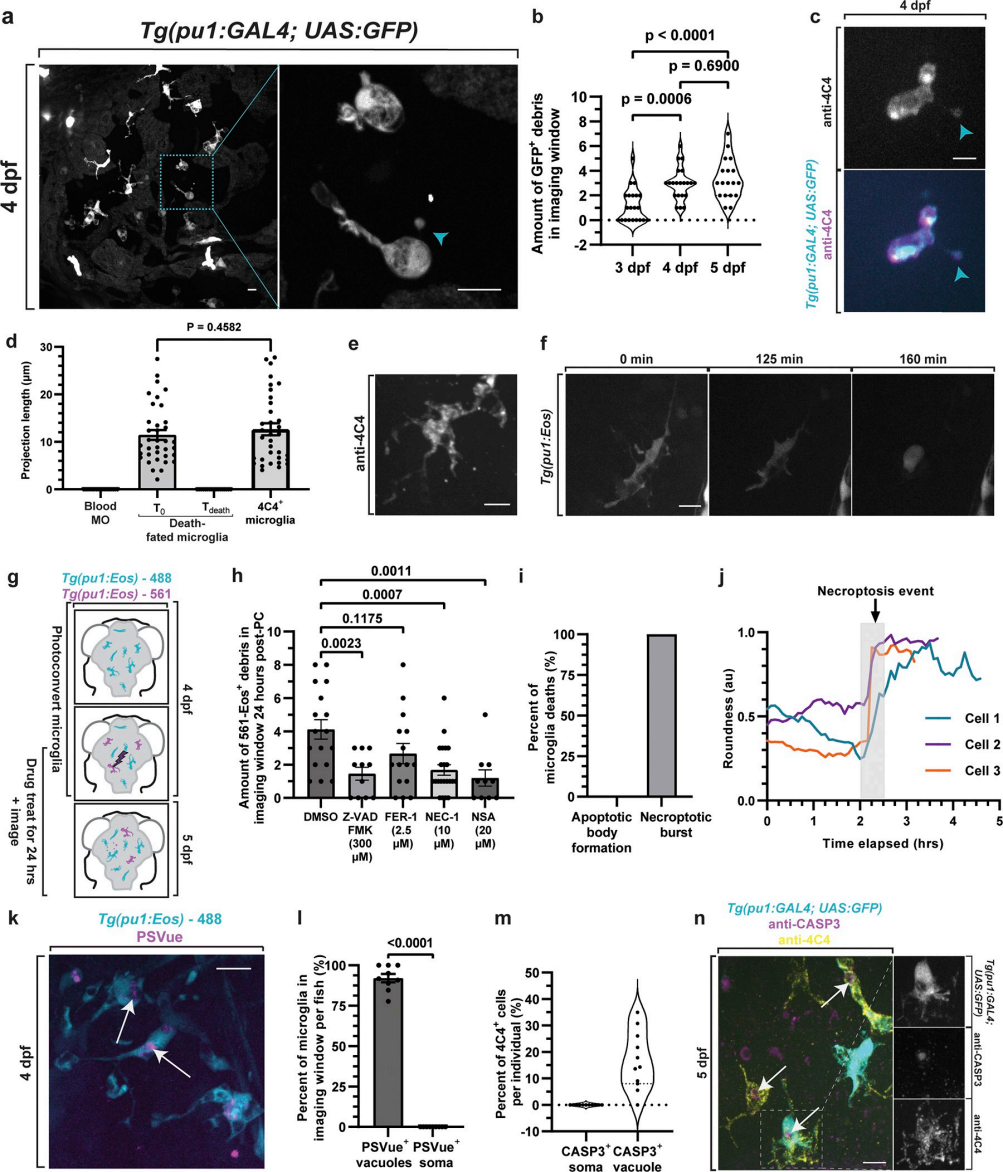
Microglia die via necroptosis in colonization stages. (a, b) Confocal images of 4 dpf *Tg(pu1*:*GFP)* brains (a) and quantification (b) showing microglia debris (blue arrowhead) and intact microglia (*p* = 0.1189 3 dpf vs. 4 dpf, *p* = 0.8368 4 dpf vs. 5 dpf, *p* = 0.0387 3 dpf vs. 5 dpf, post hoc Tukey test). (c) Confocal images of 4 dpf *Tg(pu1*:*GFP)* brains labeled with anti-4C4 demonstrating GFP^+^; 4C4^+^ debris. (d) Quantification of microglia projection length for blood macrophages (Blood MO), death-fated microglia at early time points and right before death from time-lapse movies, and of microglia labeled with 4C4 at 4 dpf (*p* = 0.4582 T_0_ death-fated microglia vs. 4C4 microglia). (e, f) Confocal image of 4C4 labeled microglia (e) and death-fated microglia (f) showing cellular morphology consistent with microglia identity at 4 dpf. (g) Schematic of experimental paradigm (g) and quantification (h) of 561-Eos^+^ debris in the brain 24-h after labeling showing Nec-1 and NSA reduce microglia debris (*p* = 0.0023 DMSO vs. Z-VAD FMK, *p* = 0.1175 DMSO vs. Fer-1, *p* = 0.0007 DMSO vs. Nec-1, *p* = 0.0011 DMSO vs. NSA, one-way ANOVA/Dunnett’s multiple-comparisons test). (i) Quantification of the percentage of microglia deaths at XX dpf in 24-h time-lapse movies that cause apoptotic bodies vs. necroptotic burst. (j) Quantification of cell roundness in a 24-h movie from 4 to 5 dpf of microglia before it dies. (k, l) Confocal images (k) and quantifications (l) of *Tg(pu1*:*Eos)* animals at 4 dpf that are treated with PsVue demonstrating PsVue labels vacuoles (arrow) but not cell bodies (*p* < 0.0001, unpaired *t* test). (m, n) Quantifications (m) and confocal images of *Tg(pu1*:*GAL4;UAS*:*GFP)* animals labeled with Casp3 and 4C4 at 5 dpf (n) showing Casp3 is located in vacuoles (arrow) but not does not label the microglia cell bodies. Scale bar is 10 μm (a, c, e, f, k, n). One-way ANOVA (b, h), *T* test (d, l). Additional supplemental material provided in S1 Fig. Descriptive statistics represented in S1 Table. The underlying data can be found in S1 Data.

To investigate how this debris could occur, we generated movies of *Tg(pu1*:*GFP)* animals for 24 h from 4 to 5 dpf. While shedding of GFP^+^ debris from living microglia is a possibility, we never saw such an event in 24-h movies. Instead, we could detect that GFP^+^ microglia debris was caused by microglia cell death (S1B Fig, *n* = 10 cells). Although our imaging used standard time-lapse imaging approaches for zebrafish, we ruled out the possibility that the death was a result of imaging parameters by scoring the amount of death in animals that were time lapsed versus not time lapsed (S1C and S1D Fig). Staining for cleaved Caspase-3 further confirmed that the imaging itself was not causing death (S1C Fig). Consistent with other reports that imaged zebrafish microglia, imaging also did not cause an increase in microglia death or alter microglia abundance (S1E, S1F, S1Q, and S1R Fig). Further, the above data represents data from single time points and time lapses, supporting the concept that the microglia death is occurring independent of imaging parameters. It is also possible that the GFP^+^ debris that is present were small cellular extensions from attached microglia but are undetectable with the 4C4 antibody or the cytosolic GFP in *Tg(pu1*:*GFP)*, but time-lapse movies at 4 dpf demonstrated stationary GFP^+^ debris puncta that clearly separated from migratory GFP^+^ cells (S1G and S1H Fig and S1 Movie). These time-lapse imaging experiments revealed that GFP^+^ debris is quickly removed from the parenchyma (S1I Fig and S1 Movie), thereby consistent with the idea that our quantifications of GFP^+^ debris is underreporting the total amount of microglia death.

Most developmental death is mediated by apoptosis [1]. To identify the molecular mechanism of this microglia death, we first treated animals with Z-VAD FMK from 4 to 5 dpf, a pan caspase inhibitor that is highly effective at blocking apoptosis in multiple cell types [22]. The Z-VAD FMK treatment was confirmed to reduce apoptosis at 5 dpf by scoring cCaspase3 in DMSO versus Z-VAD FMK (S1J Fig). To normalize the amount of microglia debris per cell, we next designed a strategy to label a specific number of microglia. To do this, we generated a *Tg(pu1*:*Eos)* animal, which expresses the photoconvertible protein, Eos, in microglia (Fig 1G) [23]. In this paradigm, we exposed 3 microglia to 405 nm light at 4 dpf and thereby photoconverted the Eos protein from green (488-Eos) to red (561-Eos) emission (S1K Fig and S2 Movie). Scoring debris in DMSO versus Z-VAD FMK-treated animals at 5 dpf was statistically different (Fig 1H) (*p* = 0.0023 DMSO (*n* = 17) versus Z-VAD-FMK (*n* = 11), Dunnett’s multiple comparisons test), indicating that the microglia death was partially dependent on apoptosis. To explore other molecular mechanisms of microglia debris, we then similarly treated animals with inhibitors of ferroptosis (Fer-1) and necroptosis (Nec-1) [24–26]. Treatment with necroptotic inhibitors, Nec-1, also reduced the amount of microglia debris while ferroptosis inhibitors did not (Fig 1H) (*p* = 0.0007 DMSO (*n* = 17) versus Nec-1 (*n* = 22), *p* = 0.0011 DMSO (*n* = 17) versus NSA (*n* = 10), Dunnett’s multiple comparisons test). To confirm our treatment of Nec-1 inhibits its well-defined Ripk1 target, we tested activation of signaling pathways downstream of Ripk1. We could not identify antibodies that label Ripk1 and MLKL specifically in zebrafish tissue. We therefore tested if Nec-1 treatment perturbed other defined Ripk1-mediated signaling by determining if NFkB activation was disrupted [27]. We treated *Tg(nfkb*:*eGFP)* which uses NFkB activation domains to drive eGFP expression and detected reduced levels of eGFP in Nec-1 versus DMSO animals at 4 dpf (S1L and S1M Fig). This reduction is specific to Ripk1 inhibition because treatment with NSA did not change *nfkb*:*eGFP* (S1L and S1M Fig). To provide a complementary approach to test the role of necroptosis, we repeated the photoconversion paradigm with NSA, an inhibitor of MLKL that is downstream of Ripk1 and specific to necroptosis. Similar to Nec-1 treatment, NSA treatment also reduced the number of debris, consistent with the idea that embryonic microglia death is dependent on necroptosis (Fig 1H). While the amount of debris decreased after Nec-1 and NSA treatment, the overall number of microglia (as labeled by 4C4) after 24 h of treatment with DMSO, Nec-1 or NSA treatments were indistinguishable (S1N Fig).

To further understand if embryonic microglia death was necroptotic or apoptotic and not just dependent on other necroptotic or apoptotic cells, we visualized microglia as they undergo death with *Tg(pu1*:*GFP)* animals in 24-h time-lapse movies from 4 to 5 dpf. We scored the dynamic features of apoptotic cellular bodies that would be consistent with apoptosis [13,15,28]. In contrast, a rounding up of a cell followed by sudden disappearance of cytoplasmic labeling would be indicative of necroptosis [29]. Tracing of individual microglia every 5 min for 24 h revealed that microglia death did not exhibit apoptotic cellular bodies (Fig 1I, *N* = 5 animals, *n* = 10 cells). Instead, most microglia that died halted their migration, retracted all of their cellular processes to reduce their area, rounded up and then shortly later, burst (Figs 1I, 1H, and S1P and S3 Movie). We further identified that microglia membranes at 4 dpf do not label with the phosphatidylserine reporter PSVue (Fig 1K and 1l, *N* = 9 animals, *n* = 92 cells), which is normally localized on apoptotic cells [30,31]. We complemented this approach with staining of cleaved-Caspase3, which is present in apoptotic cells. While cCaspase3 labeled embryonic brain cells at 4 dpf, we did not detect cCaspase3+ microglia (Fig 1M and 1N). Instead, cCaspase3 was only present within microglia phagosomes, similar to PSVue (Fig 1M and 1N). Together with the pharmacological inhibition, this strongly supports the idea that developmental microglia die via necroptosis. These experiments do not rule out that microglia could die via other cell death processes, but for this report, we focused on the necroptotic death of microglia.

To next investigate cell biological features that promote this microglia necroptosis, we tracked the lifespan of individual microglia until they died. To do this, we utilized time-lapse imaging of the zebrafish brain to track individual microglia. In 4 dpf *Tg(pu1*:*GFP)* animals, we could detect microglia with cytosolic GFP labeling that contained GFP^-^ vacuole-like structures (Fig 2A). Transgenic animals for specific cell types demonstrated the content of these vacuoles were astroglial (from *Tg(gfap*:*NTR-mCherry)*) [32], neuronal (from *Tg(nbt*:*dsRed)*) [15], and to a lesser extent oligodendroglial (from *Tg(sox10*:*mRFP)*) [33] debris (Figs 2B, S2A, and S2B). For this manuscript, we define vacuoles as a descriptive term for XFP-negative inclusions within microglia. By collecting images that spanned the brain, we identified that vacuole containing microglia represented the majority of microglia in the developing brain (S2C Fig, *N* = 5 animals). We fate mapped these microglia by imaging them in the brain every 5 min for 24 h and then tracked microglia and the vacuoles in them at every time point from 4 to 5 dpf. Plotting each individual microglia in 24-h periods demonstrated that microglia transitioned from non-vacuole containing to vacuole containing (Fig 2C and 2D). However, we could not detect microglia that transitioned from vacuole containing to non-vacuole containing over the 24-h imaging period (Fig 2C and 2E, *N* = 4 animals, *n* = 12 cells). In these movies, we instead identified that all the microglia that died contained vacuole-like structures (Fig 2C and 2F, *N* = 4 animals, *n* = 10 cells), introducing the possibility that most embryonic microglia die before they completely digest efferocytic debris. To explore the possibility that overload of cellular debris caused microglia death, we measured the amount of vacuoles and size of vacuoles in movies of microglia 5 time points before they died and compared that to microglia that did not die in our imaging window. However, we did not detect any amount of load that predicted death between death-fated microglia and living microglia (S2D and S2E Fig).

**Fig 2:**
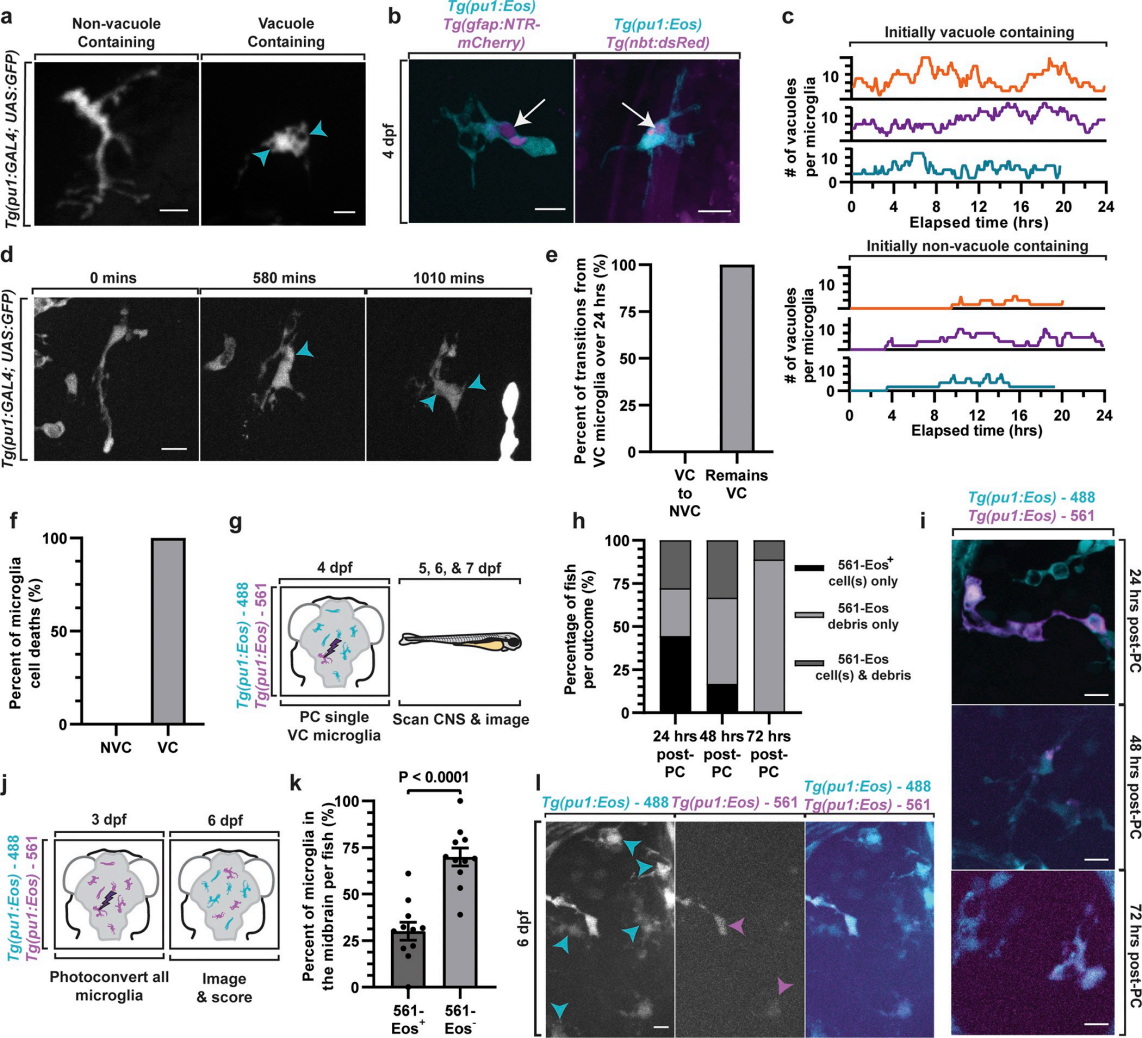
Developmental microglia have short lifespans after becoming vacuole-containing. (a) Confocal images from a 24-h time-lapse movie from 4 to 5 dpf showing microglia in *Tg(pu1*:*gfp)* animals that contain vacuoles and microglia that do not have vacuoles. Arrowhead denotes vacuoles. (b) Confocal images of *Tg(pu1*:*Eos); Tg(gfap*:*NTR-mCherry)* and *Tg(pu1*:*Eos); Tg(nbt*:*DsRed)* animals at 4 dpf demonstrating microglia that contain *gfap*^*+*^ and *nbt*^*+*^ debris. Arrow denotes debris within vacuoles. (c, d) Quantifications (c) and confocal images (d) of vacuoles inside individual microglia from 24-h time lapse from 4 to 5 dpf of *Tg(pu1*:*GFP)* animals. The number of vacuoles per microglia is scored every 5 min for 24 h. Arrowhead denotes vacuoles. (e, f) The percentage of total microglia that transition between different vacuole-containing states (e) and the state of cells before their death (f) in 24-h time-lapse movies. (g, h) Schematic of experimental design (g) and quantifications (h) to test the fate of microglia. Quantifications in (h) demonstrate the percentage of occurrences that 561-Eos^+^ microglia in *Tg(pu1*:*Eos)* results in 561-Eos^+^ intact microglia, 561-Eos^+^ microglia debris, or 561-Eos^+^ intact microglia and microglial debris 24, 48, and 72 h after labeling. (i) Confocal images demonstrating outcomes that were quantified in (h). (j) Schematic of experimental paradigm demonstrating that all microglia in the brain are labeled and then scored at 6 dpf. (k) Quantification shows the percentage of microglia at 6 dpf that are labeled with 561-Eos and negative for 561-Eos (*p* < 0.0001, unpaired *t* test). (l) Confocal images of microglia at 6 dpf in the experiment represented in (j, k). Blue arrowheads represent microglia within Eos-561 labeling. Magenta arrowheads shows Eos-561^+^ microglia scale bar is 10 μm (a, b, d, i, l). Additional supplemental material provided in S2 Fig. Descriptive statistics represented in S1 Table. The underlying data can be found in S1 Data.

There seemed to be 2 simple hypotheses for these results: (1) either the completion of the digestion phase of microglia exceeds the 24-h period or (2) developmental microglia die because they are mostly irreversibly vacuole containing. To explore these hypotheses, we tracked individual microglia over longer developmental periods. Using *Tg(pu1*:*Eos)* animals, we photoconverted a single microglia that contained vacuoles per animal at 4 dpf, then imaged single z-stacks at 1, 2, and 3 days after photoconversion in each animal (Fig 2G). After 24 h, 3 outcomes occurred: (1) 27.78% of animals had only 561-Eos^+^ debris in the brain; (2) 27.78% of animals had 561-Eos^+^ debris and 561-Eos^+^ cells that contained vacuoles; or (3) 44.44% of animals had 561-Eos^+^ cells that contained vacuoles, consistent with the idea that the microglia either died or divided (Fig 2H, *N* = 18 animals). However, we could not detect 561-Eos^+^ microglia that did not contain vacuoles; 3 days after photoconversion we rarely detected intact 561-Eos^+^ microglia, but identified 561-Eos^+^ debris in the brain (Fig 2H, *N* = 18 animals). While it is possible that detection of Eos was compromised by time or that the photoconversion paradigm promoted cell death, we confirmed with a second transgenic line, *Tg(sox10*:*Eos)*, that other long-lived, dividing cells [34,35], like dorsal root ganglia cells, could still be visualized as intact 3 days after photoconversion (S2F and S2G Fig, *N* = 8 animals, *n* = 48 cells). These data are consistent with the likelihood that once developmental microglia have vacuoles, they die within 3 days.

Microglia are thought to be long-lived cells that initially populate the brain during development [3]. Our results, however, indicate that once embryonic microglia become vacuole-containing in the zebrafish brain, they have short lifespans (Fig 2H, *N* = 18 animals). Scoring of microglia from 3 to 5 dpf also showed 76.65% ± 1.95% of microglia are vacuole-containing on a given day (S2C Fig) and thus may be expected to die. Therefore, we next explored the scale of microglia death in the embryonic brain. To do this, we photoconverted all microglia in the brain of *Tg(pu1*:*Eos)* animals at 3 dpf, and then scored the ratio of microglia that were photoconverted at 5 dpf (Fig 2J). These results indicated that on average 30.07% ± 4.81% of *pu1*:*Eos*^*+*^ cells in the brain were photoconverted (Fig 2K, *N* = 11 animals, *n* = 240 cells, S2H and S2I), supporting the idea that during peak stages of neural debris, microglia have short lifespans less than 3 days. We also expected to see a subset of 488-Eos^+^ microglia because embryonic microglia are still generated from new invading cells at this age [13]. Quantification of the number of 488-Eos+ (561-Eos-) cells supports this idea (Fig 2K). Scoring of all microglia demonstrated that the overall number of microglia also increases (S2I Fig), confirming previous findings that microglia continue to infiltrate the brain during colonization [13]. To ensure that photoconversion of all brain microglia did not impact overall population size, we scored the number of microglia in the brains of non-photoconverted and photoconverted animals at 6 dpf (S2J Fig). We found no difference in the number of microglia between photoconverted and non-photoconverted animals (S2J Fig).

These results indicated that microglia debris is present when microglia are not only expanding but also phagocytic [3,12,13,15,36–39], at least of apoptotic debris. It is unclear how necroptotic debris would be removed in the embryo. Therefore, we next asked which cell type was clearing microglia necroptotic debris. To do this, we searched the brain for microglia debris encased in the different phagocytic cells. We first assayed microglial debris in *Tg(pu1*:*RFP)*;*Tg(glast*:*GFPcaax-TA-nucRFP)* [40] and *Tg(pu1*:*GFP);Tg(sox10*:*mRFP)* at 4 dpf, which represent astroglia and neural crest/oligodendrocyte lineage cells that have been characterized as phagocytic [19,41,42]. However, we could not detect microglia debris encased in either cell type. We therefore devised a strategy to visualize microglia debris within living microglia by using our *Tg(pu1*:*Eos)-*photoconversion paradigm to label microglia before they died and were cleared at 4 dpf (Fig 1G). We then collected images of the brain 24 and 48 h after photoconversion when 561-Eos^+^ microglia debris was present. In this paradigm, we detected 561-Eos^+^ microglia debris within 488-Eos^+^ microglia of all photoconverted animals (Fig 3A–3C, *N* = 12 animals), revealing the concept of microglia cannibalism (Fig 3B).

**Fig 3:**
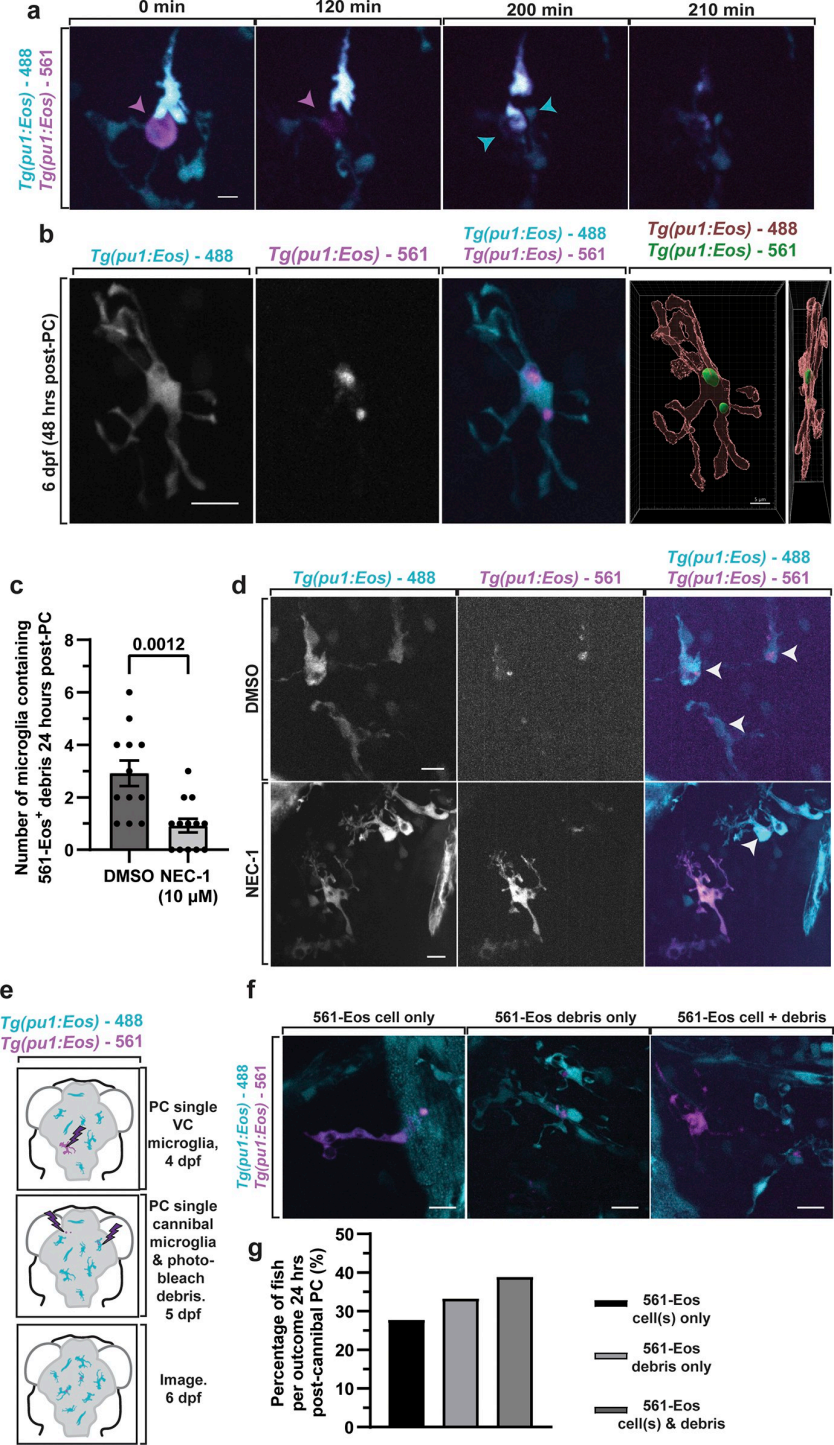
Microglia debris is cannibalized. (a) Confocal images from 24-h movie from 4 to 5 dpf of *Tg(pu1*:*Eos)* animals that had 561-Eos^+^ microglia that died and was cleared by 488-Eos^+^ microglia. Arrowheads denote a cell that undergoes death and is cannibalized. Arrowheads show microglia that dies and then is cleared by other microglia. (b) Confocal images of *Tg(pu1*:*Eos)* at 24 h form 4 to 5 dpf after labeling of 561-Eos^+^ microglia showing 561-Eos^+^ microglia debris within 488-Eos^+^ intact microglia. (c, d) Quantification (c) and confocal images (d) of the amount of microglia that contained cannibalized 561-Eos^+^ debris in *Tg(pu1*:*Eos)* animals treated with DMSO and Nec-1 for 24 h (*p* = 0.0012 DMSO vs. Nec-1, unpaired *t* test). Arrowhead denotes cannibalistic microglia. (e, f) Schematic of experimental design (e), quantifications (g), and confocal images (f) to determine the fate of cannibalistic microglia. Quantifications (g) score the fate (561-Eos^+^ intact microglia, 561-Eos^+^ microglia debris or 561-Eos^+^ intact microglia and microglial debris) of cannibalistic microglia. Scale bar is 10 μm (a, b, d, f). Additional supplemental material provided in S3 Fig. Descriptive statistics represented in S1 Table. The underlying data can be found in S1 Data.

There were 2 likely hypotheses of how cannibalism occurred: (1) Microglia clear debris from dead microglia; or (2) microglia cannibalized portions of living microglia. To distinguish between these possibilities, we again labeled a random subpopulation of microglia with 561-Eos at 4 dpf and tracked the appearance of microglia debris within migrating microglia in 24-h time-lapse movies. In these movies, the majority of cannibalism events showed a sudden disappearance of fluorescence in the dying microglia corresponding with the arrival of other microglia that instantly had 561-Eos^+^ puncta (Fig 3A). We did not detect microglia clearing of phagocytic cellular portions from other living microglia. To ensure this process was not a consequence of the photoconversion paradigm, we performed time-lapse imaging of *Tg(pu1*:*GFP)* animals for 24 h from 4 to 5 dpf. These movies revealed stationary and isolated GFP^+^ debris that rapidly disappeared when intact GFP^+^ microglia approached the debris (Figs 1F and S3A).

If microglia necroptosis was a causative event for microglia cannibalism, then inhibiting microglia death should reduce the amount of microglia cannibalism. We therefore scored the amount of cannibalism in DMSO versus Nec-1–treated animals at 5 dpf and detected that Nec-1–treated animals had less cannibalistic microglia than DMSO (Fig 3C and 3D) (*p* = 0.0012 DMSO (*N* = 12) versus NEC-1 (*N* = 13), unpaired *t* test), consistent with the hypothesis that microglia cannibalism is driven by microglia death. Taken together, this introduces a potential paradox that once developmental microglia become phagocytic, they die, which produces debris that is eventually cannibalized by other microglia—such cannibalistic microglia are then vacuole-containing and thus may be expected to die, ultimately driving turnover of embryonic microglia.

To explore this paradox, we tracked the fate of embryonic microglia that were cannibalistic. In this paradigm, we first photoconverted an individual microglia in *Tg(pu1*:*Eos)* animals at 4 dpf and then selected animals at 5 dpf that had 561-Eos^+^ debris inside 488-Eos^+^ microglia but did not contain intact 561-Eos^+^ cells. We then bleached all the 561-Eos^+^ debris in the brain of these animals, then photoconverted Eos to label the cannibalistic microglia with 561-Eos; 24 h later, these animals were then scored for the presence of 561-Eos^+^ debris and/or cells in the brain (Fig 3E–3G, *N* = 18 animals). Control animals that were not rephotoconverted demonstrated negligible levels of photoconverted-debris supporting the idea the 561-Eos was bleached in the experimental paradigm (S1B and S1C Fig). In animals where cells were photoconverted a second time, we identified 33.33% of cannibalistic microglia generated 561-Eos^+^ debris in the brain, 38.89% of cannibalistic microglia caused 561-Eos^+^ cells, and 561-Eos^+^ debris and 27.78% of cannibalistic microglia produced 561-Eos^+^ cells (Fig 3G, *N* = 18 animals), consistent with the hypothesis that most cannibalistic microglia die.

We sought to further explore the paradox that most phagocytic microglia die, leading to debris that must be cannibalized, which then produces more phagocytic microglia that are destined to die (Fig 4A). Two predictions could be made based on that hypothesis, (1) decreasing microglia phagocytosis should reduce microglia debris and cannibalism; and (2) increasing neural debris and thereby microglia phagocytosis should increase microglia debris and cannibalism. We first reduced phagocytosis by treating *Tg(nbt*:*dsRed); Tg(pu1*:*GFP)* animals with L-SOP from 4 to 5 dpf, which inhibits phosphatidylserine-dependent phagocytosis that microglia utilize to clear dead neurons [28]. Animals treated with L-SOP from 4 to 5 dpf had a reduced number of vacuole-containing microglia compared to DMSO-treated animals (Fig 4B and 4C) (DMSO (*N* = 6 animals, *n* = 132 cells), L-SOP (*N* = 6 animals, *n* = 171 cells, *p* = 0.0034 DMSO versus L-SOP, unpaired *t* test)), confirming the treatment can inhibit phagocytosis [28]. L-SOP–treated animals also had less GFP^+^ microglial debris than DMSO (Fig 4D and 4E) (*p* = 0.0005, DMSO (*N* = 6 animals, *n* = 132 cells), L-SOP (*N* = 6 animals, *n* = 171 cells, unpaired *t* test)), indicating that inhibiting phagocytosis leads to less microglia death. To test if this also reduced microglia cannibalism, we photoconverted 3 microglia in *Tg(pu1*:*Eos)* at 4 dpf, and then scored 561-Eos+ debris in 488-Eos+ microglia. Animals were either treated with water or L-SOP from 4 dpf to 5 dpf. L-SOP–treated animals had 1.125 ± 0.295 cannibalistic microglia compared to 3.000 ± 0.5345 water controls, suggesting that limiting phagocytosis reduces microglia cannibalism (*p* = 0.0072, Water (*N* = 7 animals) versus L-SOP (*N* = 8 animals), unpaired *t* test) (Fig 4F and 4G).

**Fig 4:**
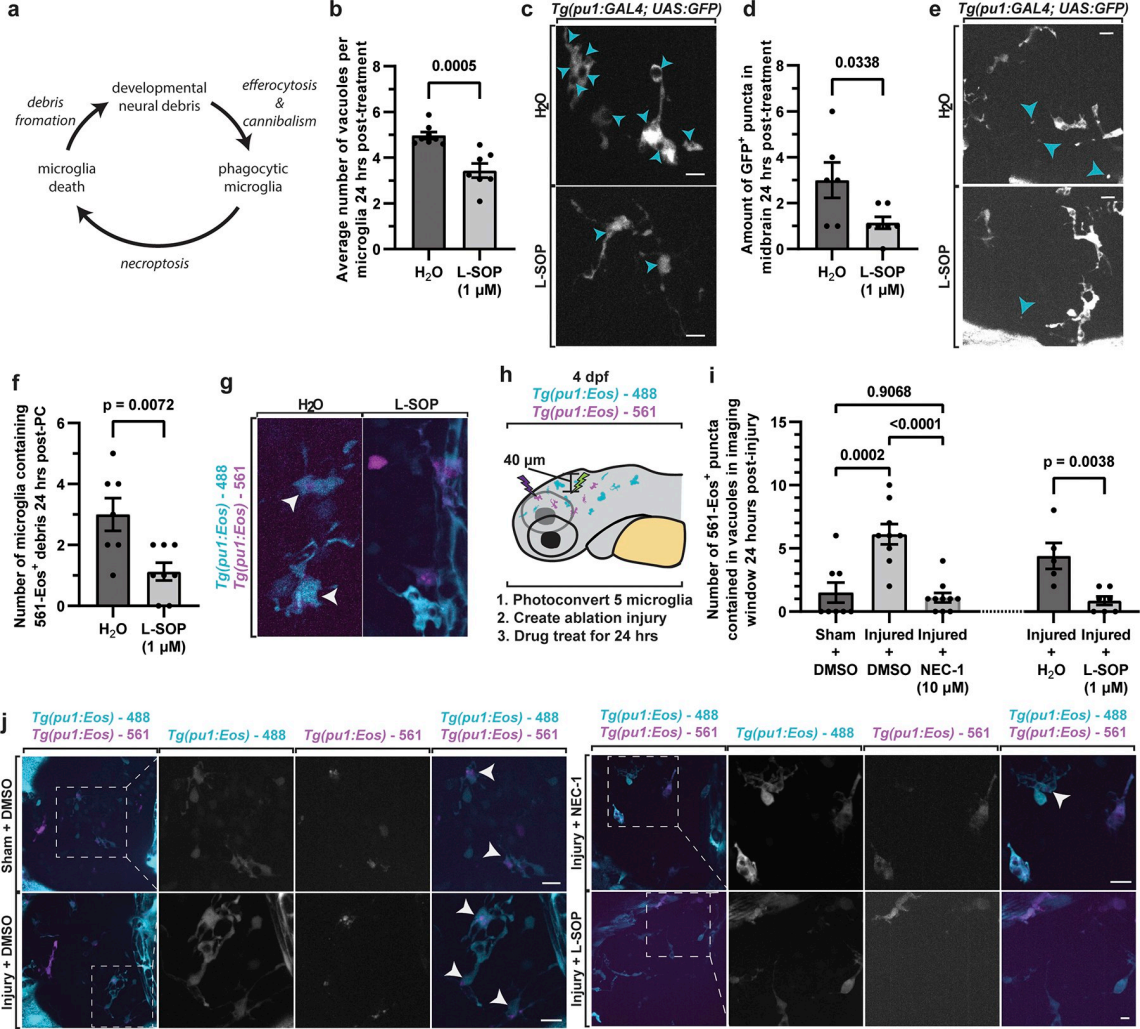
Phagocytosis and cannibalism shortens the lifespan of microglia. (a) Schematic of the paradox that involves phagocytosis, necroptosis, and cannibalism. (b) Quantifications of the average number of vacuoles in microglia at 5 dpf (*p* = 0.0338 H_2_O vs. L-SOP, unpaired *t* test). (c) Confocal images of *Tg(pu1*:*GFP)* animals at 5 dpf showing a reduction of vacuoles in microglia. Arrow denotes vacuoles. (d) Quantification of the amount of microglia debris in *Tg(pu1*:*GFP)* animals treated with H_2_O (control) or L-SOP (*p* = 0.0005, H_2_O vs. L-SOP, unpaired *t* test). (e) Confocal images demonstrating a reduction in the amount of microglia debris from (d). Arrowheads denote vacuoles. (f) Quantifications of the amount of microglia that contain 561-Eos in water for L-SOP treatments. (g) Confocal images of cannibalistic microglia at 5 dpf from treatments in (f). (h, i) Schematic of experimental design (h) and quantifications of cannibalistic microglia after laser-induced brain injury (i). Quantifications show the number of 561-Eos^+^ puncta in 488-Eos^+^ intact microglia 24 h after sham injuries or laser-injuries in *Tg(pu1*:*Eos)* animals treated with DMSO vs. Nec-1 and H_2_O vs. L-SOP for 24 h after the injury (*p* = 0.0002 Sham+DMSO vs. Injured+DMSO, *p* < 0.0001 Injured+DMSO vs. Injured+Nec-1, *p* = 0.9068 sham+DMSO vs. injured+Nec-1, post hoc Tukey test). (j) Confocal images from the experiment in (h, i) showing changes in microglia cannibalism at 5 dpf after injury in Nec-1 and L-SOP treatments. Arrowheads denote cannibalistic microglia. Scale bar is 10 μm (c, e, j). Additional supplemental material provided in S4 Fig. Descriptive statistics represented in S1 Table. The underlying data can be found in S1 Data.

To test the paradox by increasing neural debris, we created injuries in the brain to non-microglia cells to generate more neural debris that is cleared by microglia. Injuries were created with a 532 nm single-pulsed laser [16,43,44]. As a sham-injury control, the 641 nm laser was exposed to a similar size injury site. We first established that our injury paradigm created neuronal debris in *Tg(nbt*:*dsRed); Tg(pu1*:*Eos)* by scoring the amount of *dsred*^*+*^ puncta in the brain after the injury at 5 dpf (S4A Fig). Despite an average of 4.667 ± 12.69 neuronal *dsred*^*+*^ debris per imaging window, the injury paradigm did not cause detectable 488-Eos^+^ microglia debris immediately after the injury (S4A and S4B Fig) (*N* = 6 animals, *p* < 0.0001, dsRed^+^ debris versus Eos^+^ debris, unpaired *t* test). Consistent with the expansion of microglia after injury, 24 h later we visualized an increase in microglia compared to sham-injured animals (S4C Fig) (Sham (*N* = 5 animals) versus Injury (*N* = 4 animals), *p* = 0.0576, unpaired *t* test). Having established the injury paradigm, we repeated it in *Tg(pu1*:*Eos)* animals that had 5 microglia in the brain photoconverted and thereby labeled with 561-Eos^+^ at 4 dpf (Fig 4H). After 24 h, we quantified that the number of 561-Eos^+^ microglia debris present in the brain was in higher abundance in injured animals compared to sham-injured animals (Fig 4I and 4J) (Sham/DMSO (*N* = 8) versus Injury/DMSO (*N* = 9), *p* = 0.0002, post hoc Tukey test). The vast majority of this microglia debris was cannibalized by 488-Eos^+^ microglia.

If the increase in microglia phagocytosis of neuronal debris is causing microglia to die, and thereby causing more microglia cannibalism, then blocking microglia death or microglia phagocytosis should reduce the amount of microglia debris and microglia cannibalism. We, therefore, first tested if Nec-1 treatment reduced the number of cannibalistic microglia in the injury paradigm. In Nec-1–treated animals, the amount of 561-Eos^+^ microglia debris at 5 dpf returned to the level of sham-injured animals (Fig 4I and 4J) (Injury/Nec1 (*N* = 10) versus Injury/DMSO (*N* = 9), *p* < 0.0001, post hoc Tukey test). Microglia cannibalism also returned to the level of the uninjured animals, consistent with the hypothesis that phagocytic microglia death and cannibalism is impacted by necroptosis. One caution in the interpretation of the Nec-1 treatment results is that necroptotic microglia could be a direct consequence of the injury and not secondary to an increase in phagocytic microglia. To distinguish between such possibilities, we repeated the paradigm in animals treated with L-SOP from 4 to 5 dpf. In L-SOP–treated animals that were injured, 0.8571 ± 0.8997 561-Eos^+^ puncta were present in vacuoles at 5 dpf, compared to 4.400 ± 1.030 in control animals (*p* = 0.0038, unpaired *t* test H_2_O (*N* = 5 animals) versus L-SOP (*N* = 7 animals)) (Fig 4I and 4J). The simplest explanation for this collective data is that efferocytosis by microglia results in a shorted lifespan via necroptosis.

Together, our data shows that microglia that contain phagocytic debris in vacuoles have short lifespans that rarely exceed 3 days. Upon their death, developmental microglia debris is cannibalized by other microglia. The phagocytic nature of cannibalistic microglia then leads to their death and more debris that must be phagocytosed. This paradoxical process thereby establishes a cycle. In development, the paradox could be started by the apoptotic death of neural cells, which are cleared by microglia. This hypothesis may explain why inhibition of apoptosis and necroptosis reduces microglia death even though we could not detect apoptotic-based death in our imaging. This work, with others, also establishes that microglia debris is cleared by astrocytes and microglia in specific contexts [19]. Beyond the universal principle that cells overexpand during development and then die via apoptosis [1,2], this work reveals an additional mechanism that produces developmental debris, induced by sacrificial microglia that function to clear apoptotic debris. This work further introduces an additional attribute of microglia expansion in the embryo. In addition to previous studies that demonstrated microglia expansion is balanced by infiltration of progenitors and cell division of existing microglia [13], the death of microglia could also contribute to the overall abundance of microglia in the embryo. Future studies that investigate how these different attributes of the embryonic microglia pool are connected will be important. Given the data here and evidence of necroptotic microglia in disease contexts [17,18], it will be intriguing to investigate if microglia cannibalism and a subsequent paradox that is driven by microglia cell death, is a hallmark of neuropathologies through ages.

## Limitations to results

This work supports the idea that microglia die via necroptosis but does not rule out the possibility that additional modes of cell death contribute to microglia death. We use the term vacuoles above to describe a feature in microglia. These vacuoles contain debris from different neural cells and thus likely represent phagosomes. However, without testing specific phagosome markers to confirm that and to avoid giving them a biological function that was not tested (i.e., phagosome and lysosome), we elected to describe them with a more descriptive term, vacuoles. Future work will need to investigate the deeper molecular mechanism and biological significance of microglia cannibalism.

## Methods

### Ethics statement

Experimental procedures followed the NIH guide for the care and use of laboratory animals. The University of Notre Dame IACUC which is guided by the United States Department of Agriculture, the Animal Welfare Fact (USA) and the Assessment and Accreditation of Laboratory Animal Care International approved all animal studies under protocol 22-07-7322 to Cody J. Smith.

### Contact for reagent and resource sharing

All data collected for the study are included in the figures and supplemental data. Reagents are available from the authors upon request.

### Experimental model and subject details

Zebrafish strains in this study include: AB, *Tg(pu1*:*GAL4*, *UAS*:*GFP)*^*zf149*,^ [15], *Tg(gfap*:*NTR-mCherry*)^sc059,^ [32,45], *Tg(nbt*:*dsRed)*^*zf148*,^ [15], *Tg(pu1*:*Eos)*^*nt200*,^ [23], *Tg(sox10*:*Eos)*^*w9*,^ [34], *Tg(sox10*:*mRFP)*^*vu234*,^ [33], *Tg(slc1a3b*:*myrGFP-P2A-H2AmCherry*) [40], *Tg(pu1*:*GAL4*, *UAS*:*RFP)*^*hdb2*,^ [15], *Tg(nfkb*:*eGFP)* [46]. Only germline transgenic lines were used in this study. To produce embryos, pairwise matings were used. Animals were raised at 28°C in egg water in constant darkness and staged by hours or days post fertilization (hpf and dpf), confirmed by observation of developmental milestones [47]. Embryos were used for all experiments.

## Method details

### In vivo imaging

Animals were anesthetized using 3-aminobenzoic acid ester (Tricaine), covered in 0.8% low-melting point agarose, and mounted dorsally in glass-bottomed 35 mm Petri dishes [48]. A spinning disk confocal microscopes custom built by 3i technology (Denver, Colorado, United States of America) that contains: Zeiss Axio Observer Z1 Advanced Mariana Microscope, X-cite 120LED White Light LED System, filter cubes for GFP and mRFP, a motorized X,Y stage, piezo Z stage, 20X Air (0.50 NA), 63X (1.15NA), 40X (1.1NA) objectives, CSU-W1 T2 Spinning Disk Confocal Head (50 μm) with 1X camera adapter, and an iXon3 1Kx1K EMCCD or Teledyne Prime 95B cMOS camera, dichroic mirrors for 446, 515, 561, 405, 488, 561, 640 excitation, laser stack with 405 nm, 445 nm, 488 nm, 561 nm, and 637 nm with laser stack FiberSwitcher, photomanipulation from vector high speed point scanner ablations at diffraction limited capacity, Ablate Photoablation System (532 nm pulsed laser, pulse energy 60J at 200 HZ) was used to acquire images [48]. Images in time-lapse microscopy were collected every 5 min for 24 h. Images were processed with Adobe Illustrator, ImageJ, and IMARIS. Only brightness and contrast were adjusted and enhanced for images represented in this study.

### Immunohistochemistry

The primary antibody used in the confirmation of microglia debris in the brain was anti-cleaved Caspase3 (1:700, BD Biosciences) and anti-4C4 (1:50, mouse, Seiger, Becker and Becker Laboratories) [20]. The secondary antibody used was Alexa Fluor 647 goat anti-mouse (1:600, Thermo Fisher, A-21235). Staining was performed using the protocol by Nichols and Smith [48]. Larvae were fixed at 3 dpf, 4 dpf, and 5 dpf in fresh 4% paraformaldehyde in 0.1% PBS Triton-X.

### Photoconversion experiments

#### Single-cell photoconversions

*Tg(pu1*:*Eos)* embryos were grown to 4 dpf. Pre-conversion confocal *z*-stack images were taken in the 488 nm/GFP filter set and 561 nm/RFP filter set to confirm there was no nonspecific photoconversion. Single, vacuole-containing *pu1*^*+*^ cells were then photoconverted using a 5-ms 405-nm-laser pulse guide to the midbrain with mVector (3i) in the midbrain at 4 dpf following the single-cell photoconversion protocol described in Green and Smith [49]. Following photoconversion, confocal *z*-stack images were taken in the 488 nm/GFP filter set and 561 nm/RFP filter set to confirm expression of the converted Eos^+^ protein. Photoconverted animals were then grown to and imaged at 5 dpf, 6 dpf, and 7 dpf. Confocal 140 μm *z*-stack images were taken of the dorsally mounted midbrains and the trunks of the fish were manually scanned for presence of 561-Eos^+^ cells and debris. We categorized the fish based on the presence of 561-Eos^+^ debris and 561-Eos^+^ cells.

#### Cannibal tracking

*Tg(pu1*:*Eos)* embryos were grown to 3 dpf. Pre-conversion confocal *z*-stack images were taken in the 488 nm/GFP filter set and 561 nm/RFP filter set to confirm there was no nonspecific photoconversion. Single, vacuole containing *pu1*^*+*^ cells were then photoconverted using a 5-ms 405-nm-laser pulse in the midbrain at 3 dpf following the single-cell photoconversion protocol above. Following photoconversion, post-conversion confocal *z*-stack images were taken in the 488 nm/GFP filter set and 561 nm/RFP filter set to confirm expression of the converted Eos^+^ protein. Photoconverted animals were then grown to and imaged at 4 dpf. Confocal 140 μm *z*-stack images were taken of the dorsally mounted midbrains and the trunks of the fish were manually scanned for presence of 561-Eos^+^ cells and debris. Fish with 561-Eos^+^ cells at 4 dpf were removed from the experiment. Cannibal microglia were identified as 488-Eos^+^ microglia containing 561-Eos^+^ debris in the remaining fish. One cannibal microglia was then selected per fish and photoconverted. All other 561-Eos^+^ debris present was photobleached using as many 5-ms 561-nm-laser pulses as necessary. Fish were grown to and imaged at 5 dpf, 6 dpf, and 7 dpf. We categorized the fish based on the presence of 561-Eos^+^ debris and 561-Eos^+^ cells. To confirm the bleaching distinguished the 561^+^-Eos signal, a subset of animals was photobleached but not rephotoconverted.

### Injury

*Tg(nbt*:*dsRed);Tg(pu1*:*Eos)* and *Tg(pu1*:*Eos)* 4 dpf animals were anesthetized using 0.02% 3-aminobenzoic acid ester (Tricaine) in egg water. Fish were then dorsally mounted in 0.8% low-melting point agarose solution, arranged laterally on a 10 mm glass-coverslip–bottom Petri dish, and placed on the microscope anterior to posterior. Injured were performed in the midbrain. Specific site of laser-induced injury was determined by bringing the skin of the fish above the brain into focus and using the piezo Z stage to move 40 μm below the surface of the skin. This area was marked and brought into a focused ablation window. Upon focusing the targeted region, we double-clicked on a *dsRed*^*+*^ region using a 4 μm cursor tool. All laser parameters used are specific to our confocal microscope. Specific parameters include Laser Power (40), Raster Block Size (1), Double-Click Rectangle Size (120), and Double-Click Repetitions (4). After injury, fish were released from the agarose and treated with 1% DMSO or Nec-1. Sham-injured fish were followed the same procedure but were expose to single pulses of 561 nm laser with mvector rather than the Ablate! Laser.

### Chemical treatments

#### Cell death inhibitors

The chemical reagents used were Z-VAD-FMK [22], Ferrostatin-1 (Fer-1; Sigma, SML0583) [26], Necrostatin-1 (Nec-1; MedChemExpress LLC, HY-15760) [25], Necrosulfamide (NSA; Tocris Bioscience) [24]. Stock solutions of 20 mM Z-VAD-FMK, 2.5 mM Fer-1, 10 mM Nec-1, and 10 mM NSA were stored at −80°C dissolved in DMSO. Working solutions were diluted with PTU to 300 μm for Z-VAD-FMK treatments, 2.5 μm for Fer-1 treatments, 10 μm for Nec-1 treatments [50], and 20 μm for NSA treatments. All embryos were incubated in egg water until 24 hpf and incubated with PTU until desired treatment time. Fish were treated at 4 dpf, immediately after photoconversions. Control fish were incubated with 1% DMSO in PTU.

#### L-SOP treatment

The chemical reagent used for this study was O-Phospho-L-serine (L-SOP; Sigma, P0878-10MG). Stock solutions were dissolved in H_2_O to a concentration of 1 mM. Working solutions were diluted to 1 μm with PTU [28]. All embryos were incubated in egg water until 24 hpf and incubated with PTU until desired treatment time. Fish were bathed at 4 dpf with 1 μm L-SOP dissolved in egg water for 24 h. Control fish were incubated with H_2_O.

#### PSVue labeling

For imaging determining if microglia express phosphatidyl serine, embryos were bathed in a 1:250 solution of PSVue 643 (PSVue; Polysciences) diluted in egg water for 1 h prior to imaging.

### Quantification and statistics

3i Slidebook software was used to generate composite z-stack images of microglia. All individual z-stack images were sequentially observed. IMARIS (Notre Dame Imaging Core) was used to create 3D surface renderings of microglia. All graphical data represent both the mean and individual values used in each experiment unless otherwise noted. All quantifications were performed using various plug-ins available in FIJI (ImageJ) and Microsoft Excel. GraphPad Prism (version 8) software was used to perform all statistical analysis.

No statistical methods were used to predetermine sample sizes; however, all sample sizes are informed by previous publications. All statistical tests were run with biological replicates, not technical replicates. Healthy animals were randomly selected for experiments. No data points were excluded from analysis. Data distribution was assumed to be normal, but this was not formally tested. Unless otherwise indicated, data collection and analysis were performed blind to the conditions of the experiments. Each experiment was repeated at least twice with similar results.

#### Quantification of debris

GFP^+^ debris, 488-Eos^+^ debris, 561-Eos^+^ debris, *gfap*^+^ debris, *nbt*^+^ debris, and *sox10*^+^ debris were counted manually in Slidebook and ImageJ across all consecutive images in *z*-stacks of the midbrain. 488-Eos^+^ microglia were considered cannibalistic if they contained 561-Eos^+^ debris.

#### Quantification of vacuoles

The 24-h time-lapse movies were taken of 4 dpf *Tg(pu1*:*GAL4; UAS*:*GFP)* fish. Individual microglia were manually tracked at every 5-min time point of the time lapses. At each time point, the number of vacuoles an individual microglia contained was counted by going through images in *z*-stacks and was supported by the use of Slidebook’s 4D volume view feature. Vacuoles were counted at every time point until the microglia left the capture window. Vacuoles were defined as GFP^-^ inclusions of any size that were completely contained within GFP^+^ microglia.

#### Cell morphology quantifications

Analysis of microglia area and roundness were used to describe changes in microglia morphology. Microglia area and roundness were measured at every 5-min time point before death using the trace feature on ImageJ. ImageJ calculates roundness using the following formula:

$$Roundness=\frac{4\times area}{\pi \times {\left(major\;axis\right)}^{2}}$$

#### IMARIS

3D surface reconstructions were generated using IMARIS. The surface tool was use to generate surface renderings from confocal stacks taken with 1 μm step sizes. Only brightness and contrast were adjusted.

### Softwares

ImageJ and Slidebook were used to produce and process confocal images. Graphpad prism was used to generate all graphs and statistical analysis. Adobe Illustrator was used to compile the figures and p1.

## Supporting information

S1 Fig Supplemental material for Fig 1. (a) Quantification of microglia projection abundance for blood macrophages (Blood MO), death-fated microglia at early time points and right before death from time-lapse movies from 4 to 5 dpf, and of microglia labeled with 4C4 at 4 dpf (*p* = 0.1437 T0 death-fate microglia vs. 4C4 microglia). (b) Quantification of the amount of debris causing events that result from shedding vs. cell death in 24-h time-lapse movies of 4 dpf *Tg(pu1*:*GFP)* animals. (c) Quantification of CASP3+ cells in animals that were time lapses for 24 h vs. not time lapsed from 4 to 5 dpf (*p* = 0.8728 time lapsed vs. not time lapsed). (g) Confocal images from a 24-h time lapse from 4 to 5 dpf of *Tg(pu1*:*GFP)* animals demonstrating debris that is distinct from intact microglia (blue arrowhead) that disappears after intact microglia (magenta arrowhead) migrates across it. (h) Quantifications from (d) demonstrating that *pu1*^*+*^ debris is not migratory like *pu1*^*+*^ cells and thereby not physically connected. (i) Quantification from a time lapse from 4 to 5 dpf of *Tg(pu1*:*GFP)* (microglia death) and *Tg(nbt*:*dsRed)* (neuronal death) animals showing how quickly debris is cleared in the brain. (j) Quantification of the number of CASP3+ cells in animals treated with DMSO vs. Z-VAD (*p* = 0.0002). (k) Images from a confocal microscope of 4 dpf *Tg(pu1*:*Eos)* animals before and after exposure to 405 nm. Note that absence of *Tg(pu1*:*Eos)* -561^*+*^ before photoconversion. (l) Confocal images of *Tg(nfkb*:*GFP)* animals at 5 dpf stained with 4C4 after treatment for 24 h of DMSO, NEC-1, and NSA. (m) Quantification of the percent of *nfkb*+; 4C4+ microglia in *Tg(nfkb*:*GFP)* animals at 5 dpf stained with 4C4 after treatment for 24 h of DMSO, NEC-1, and NSA (*p* < 0.0001 DMSO vs. NEC-1, *p* = 0.9540 DMSO vs. NSA). (n) Quantification of the abundance of microglia (n) and number of vacuoles in those microglia (o) of 5 dpf animals treated for 24 h with DMSO, NEC-1, and NSA. (p) Quantification of the area of microglia during cell death event from 4 to 5 dpf. (q, r) Confocal images of *Tg(pu1*:*GFP)* animals (q) and quantifications of microglia abundance (r) at time point 0 h and 24 h from a 24-h time lapse and non-time lapsed animals demonstrating that the number of microglia is not impacted by 24-h time lapses. Note that cells rapidly decrease area just before the necroptosis event. Scale bar is 25 μm (d, e). Scale bar is 10 μm (k, l). Descriptive statistics represented in S1 Table. The underlying data can be found in S1 Data.
(TIF)

S2 Fig Supplemental material for Fig 2. (a) Quantification of the percentage of vacuoles in microglia that enclose each type debris from *Tg(gfap*:*NTR-mCherry)*, *Tg(nbt*:*dsRed)*, and *Tg(sox10*:*mRFP)* animals at 4 dpf (*p* = 0.9589 *gfap* vs. *nbt*, *p* < 0.0001 *gfap* vs. *sox10*, *p* < 0.0001 *nbt* vs. *sox10*, post hoc Tukey test). (b) Confocal images of *Tg(pu1*:*Eos); Tg(sox10*:*mRFP)* animals at 4 dpf showing *sox10*^*+*^ debris within *pu1*^*+*^ microglia. (c) Quantification of the percentage of microglia per 4 dpf animals that are non-vacuole (NVC) vs. vacuole containing (VC) (*p* < 0.0001 NVC vs. VC, Fisher’s exact test). (d) Quantification of the diameter of microglia vacuoles from 24-h time-lapse movies of *Tg(pu1*:*GFP)* animals at 4 dpf (death-fated). Such microglia were compared to microglia that were labeled with 4C4 at the corresponding age (*p* = 0.5912, *t* test). (e) Confocal images of death-fated microglia and microglia stained with 4C4 that were used to generate (d). (f) Confocal images of *Tg(sox10*:*Eos)* animals that were photoconverted at 4 dpf and imaged at 4 and 7 days, demonstrating the Eos photoconversion is stably detected at least 3 days after photoconversion. (g) Quantifications from (f) that demonstrate that photoconversion causes stable labeling of Eos^+^ cells. (h) Quantification of the abundance of 561-Eos^+^ microglia in *Tg(pu1*:*Eos)* animals that had all microglia photoconverted a 3 dpf and then quantified at 6 dpf. Each data point with connect line represent a single animal. Note the decrease in 561-Eos+ microglia. (i) Quantification of animals depicted in (h) and total number of microglia is quantified. Note that the overall abundance of microglia increases. (j) Quantification of number of microglia in photoconverted (+PC) and non-photconverted (-PC) animals at 6 dpf (*p* = 0.8665, *t* test). Scale bar is 10 μm (b, e, f). Descriptive statistics represented in S1 Table. The underlying data can be found in S1 Data.
(TIF)

S3 Fig Supplemental material for Fig 3. (a) Quantification of the abundance of 561-Eos^+^ debris in *Tg(pu1*:*Eos)* animals that had microglia photoconverted at 4 dpf, and then the debris was photobleached at 5 dpf (photo-bleach) and images were captured at 6 dpf. This is in contrast to the quantification of 561-Eos^+^ debris from animals that had microglia photoconverted at 4 dpf, photobleached at 5 dpf, rephotoconverted at 5 dpf, and then imaged at 6 dpf (*p* < 0.0001, *t* test). (b) Confocal images from the experiment and quantification depicted in (a). Scale bars are 25 μm (b). Descriptive statistics represented in S1 Table. The underlying data can be found in S1 Data.
(TIF)

S4 Fig Supplemental material for Fig 4. (a) Schematic demonstrating how injuries were created and calibrated in animals. (b) Quantification of the amount of debris immediately after injury at 4 dpf from Tg(*nbt*:*DsRed)*^*+*^ vs. *Tg(pu1*:*Eos)*^*+*^ cells in the injury paradigm (*p* < 0.0001 dsRed^+^ vs. 488-Eos^+^, unpaired *t* test). (c) Quantification of the percentage increase in *Tg(pu1*:*Eos)*^*+*^ cells in animals 24 h after sham or injury (*p* = 0.0576 sham vs. injury, unpaired *t* test). Note the microgliosis consistent with focal brain injuries. Descriptive statistics represented in S1 Table. The underlying data can be found in S1 Data.
(TIF)

S1 Table Table of descriptive statistics. Table that presents the descriptive statistics for every figure panel that presents quantifications.
(XLSX)

S1 Movie Microglia death in the intact brain, example1. Confocal images from a 24-h time lapse starting at 4 dpf of *Tg(pu1*:*GFP)* animals showing microglia death and quick removal of that debris.
(AVI)

S2 Movie Microglia death in the intact brain, example2. Confocal images from a 24-h time lapse starting at 4 dpf of *Tg(pu1*:*GFP)* animals showing microglia death and quick removal of that debris.
(AVI)

S3 Movie Microglia debris is separate from intact microglia. Confocal images from a 24-h time lapse starting at 4 dpf of *Tg(pu1*:*GFP)* animals demonstrating that GFP^+^ debris is stationary while intact GFP^+^ microglia migrate within the brain. Note that the debris disappears after an intact microglia migrates by it.
(AVI)

S4 Movie Precise photoconversion of single cells. Time-lapse video of single confocal slices in *Tg(pu1*:*Eos)* animals during the photoconversion process. Note that the single microglia is only 488 nm Eos (green) but then is converted to 561 nm Eos (magenta).
(AVI)

S5 Movie Microglia cannibalism. Confocal images of a 24-h time lapse of 4 dpf *Tg(pu1*:*Eos)* animals that had photoconverted microglia demonstrating a 561-Eos^+^ cell that dies and is cannibalized by 488-Eos^+^ cell.
(AVI)

S6 Movie Time-lapse imaging of zebrafish brain for 24 h, example 1. Representative movie of *Tg(pu1*:*gal4-uas*:*GFP)* animals starting at 4 dpf for 24 h showing that microglia behave normally while imaging. Cell death are marked with a circle 10 time points before death and a square for 10 time points following death. Note that cell death does not occur at a single time point but rather throughout the 24-h movie.
(AVI)

S7 Movie Time-lapse imaging of zebrafish brain for 24 h, example 1. Representative movie of *Tg(pu1*:*gal4-uas*:*GFP)* animals starting at 4 dpf for 24 h showing that microglia behave normally while imaging. Cell death are marked with a circle 10 time points before death and a square for 10 time points following death. Note that cell death does not occur at a single time point but rather throughout the 24-h movie.
(AVI)

S8 Movie Time-lapse imaging of zebrafish brain for 24 h, example 1. Representative movie of *Tg(pu1*:*gal4-uas*:*GFP)* animals starting at 4 dpf for 24 h showing that microglia behave normally while imaging. Cell death are marked with a circle 10 time points before death and a square for 10 time points following death. Note that cell death does not occur at a single time point but rather throughout the 24-h movie.
(AVI)

S1 Data Primary data for all quantifications represented in the manuscript. (XLSX)
