# Supplementary figures and images for: Microglia cannibalism and efferocytosis leads to shorter lifespans of developmental microglia
Source: PLoS Biol. 2024 Oct 30;22(10):e3002819. doi: 10.1371/journal.pbio.3002819 (PMC11524473; doi:10.1371/journal.pbio.3002819)

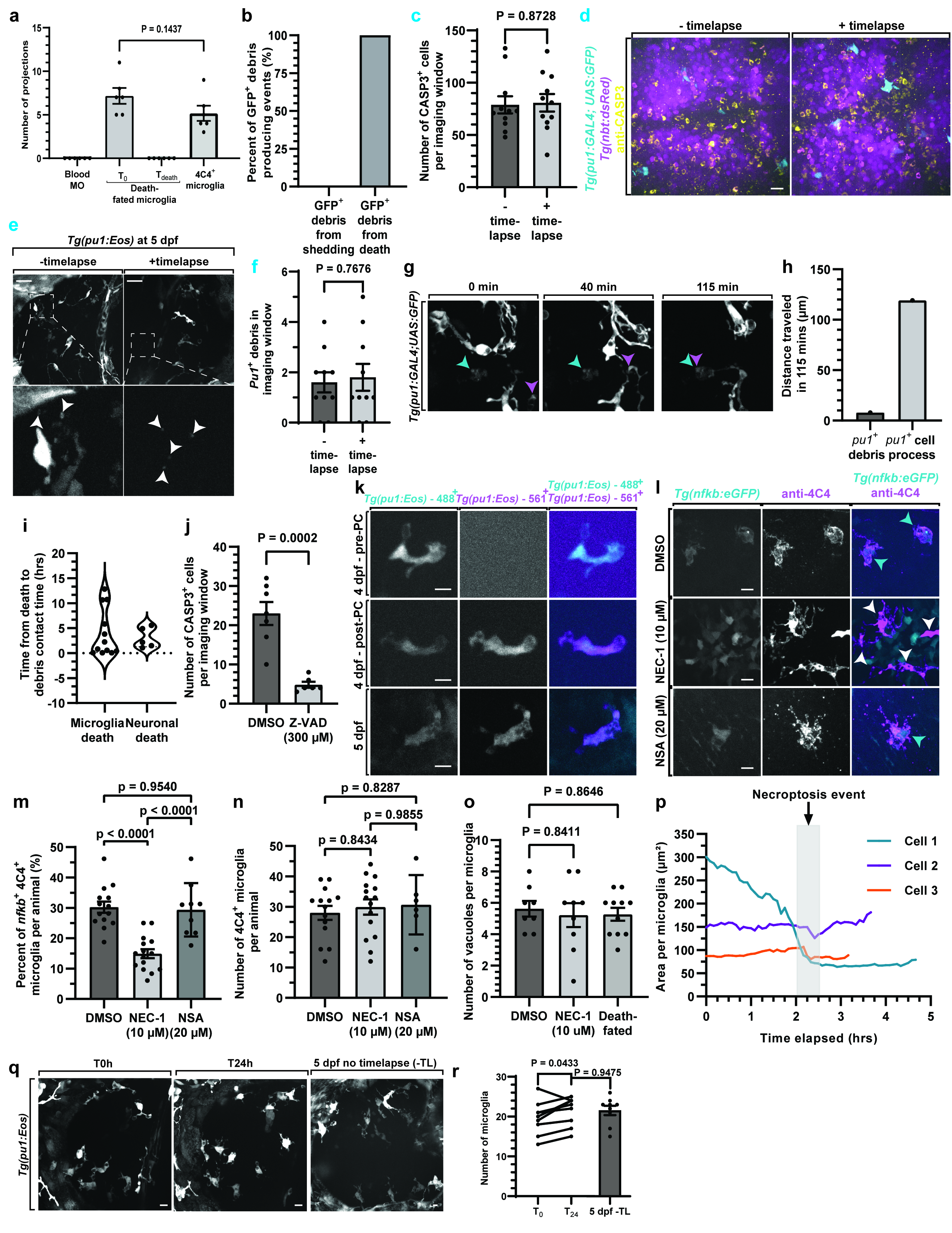

Supplement: S1 Fig — (a) Quantification of microglia projection abundance for blood macrophages (Blood MO), death-fated microglia at early time points and right before death from time-lapse movies from 4 to 5 dpf, and of microglia labeled with 4C4 at 4 dpf (p = 0.1437 T0 death-fate microglia vs. 4C4 microglia). (b) Quantification of the amount of debris causing events that result from shedding vs. cell death in 24-h time-lapse movies of 4 dpf Tg(pu1:GFP) animals. (c) Quantification of CASP3+ cells in animals that were time lapses for 24 h vs. not time lapsed from 4 to 5 dpf (p = 0.8728 time lapsed vs. not time lapsed). (g) Confocal images from a 24-h time lapse from 4 to 5 dpf of Tg(pu1:GFP) animals demonstrating debris that is distinct from intact microglia (blue arrowhead) that disappears after intact microglia (magenta arrowhead) migrates across it. (h) Quantifications from (d) demonstrating that pu1+ debris is not migratory like pu1+ cells and thereby not physically connected. (i) Quantification from a time lapse from 4 to 5 dpf of Tg(pu1:GFP) (microglia death) and Tg(nbt:dsRed) (neuronal death) animals showing how quickly debris is cleared in the brain. (j) Quantification of the number of CASP3+ cells in animals treated with DMSO vs. Z-VAD (p = 0.0002). (k) Images from a confocal microscope of 4 dpf Tg(pu1:Eos) animals before and after exposure to 405 nm. Note that absence of Tg(pu1:Eos) -561+ before photoconversion. (l) Confocal images of Tg(nfkb:GFP) animals at 5 dpf stained with 4C4 after treatment for 24 h of DMSO, NEC-1, and NSA. (m) Quantification of the percent of nfkb+; 4C4+ microglia in Tg(nfkb:GFP) animals at 5 dpf stained with 4C4 after treatment for 24 h of DMSO, NEC-1, and NSA (p < 0.0001 DMSO vs. NEC-1, p = 0.9540 DMSO vs. NSA). (n) Quantification of the abundance of microglia (n) and number of vacuoles in those microglia (o) of 5 dpf animals treated for 24 h with DMSO, NEC-1, and NSA. (p) Quantification of the area of microglia during cell death event from 4 to 5 dpf [file pbio.3002819.s001.tif]

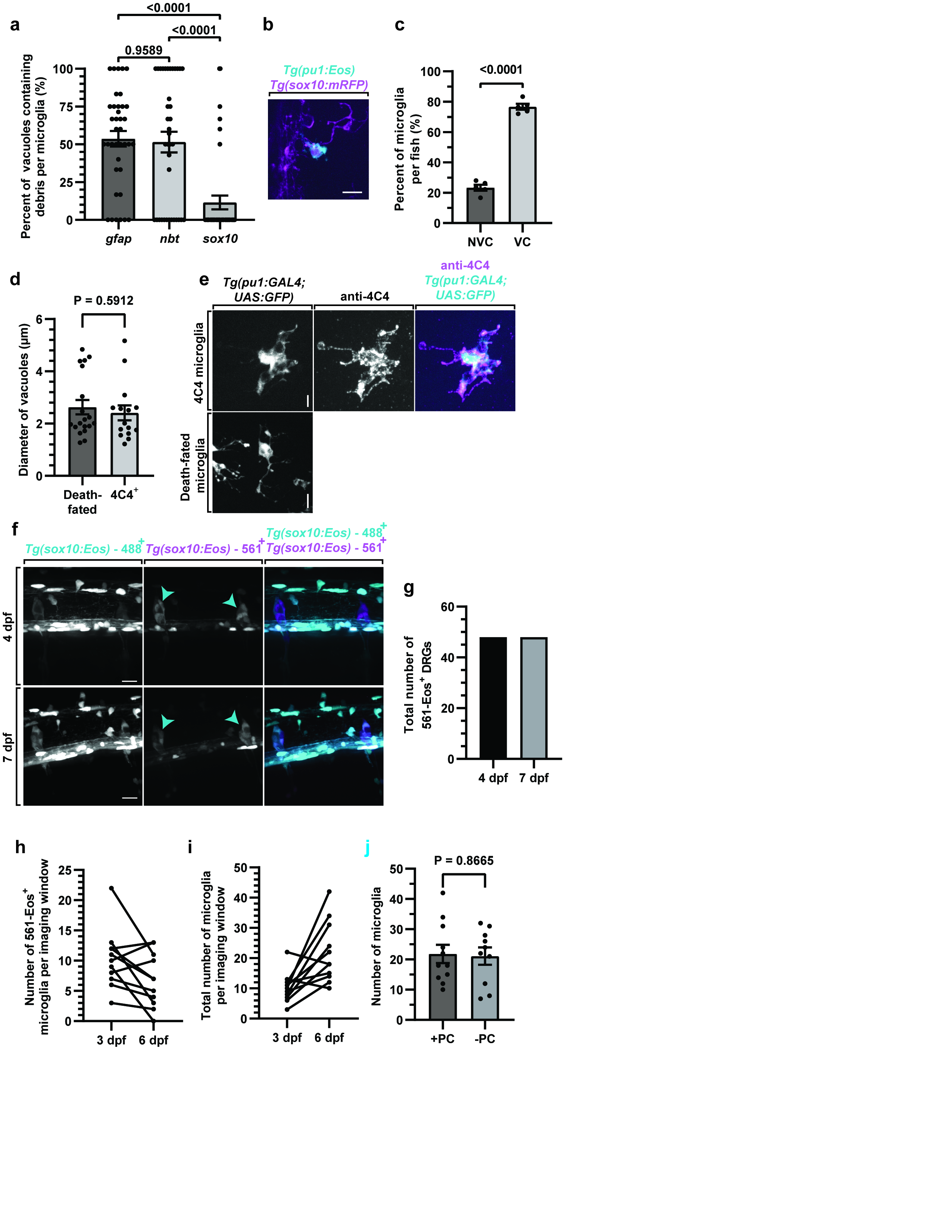

Supplement: S2 Fig — (a) Quantification of the percentage of vacuoles in microglia that enclose each type debris from Tg(gfap:NTR-mCherry), Tg(nbt:dsRed), and Tg(sox10:mRFP) animals at 4 dpf (p = 0.9589 gfap vs. nbt, p < 0.0001 gfap vs. sox10, p < 0.0001 nbt vs. sox10, post hoc Tukey test). (b) Confocal images of Tg(pu1:Eos); Tg(sox10:mRFP) animals at 4 dpf showing sox10+ debris within pu1+ microglia. (c) Quantification of the percentage of microglia per 4 dpf animals that are non-vacuole (NVC) vs. vacuole containing (VC) (p < 0.0001 NVC vs. VC, Fisher’s exact test). (d) Quantification of the diameter of microglia vacuoles from 24-h time-lapse movies of Tg(pu1:GFP) animals at 4 dpf (death-fated). Such microglia were compared to microglia that were labeled with 4C4 at the corresponding age (p = 0.5912, t test). (e) Confocal images of death-fated microglia and microglia stained with 4C4 that were used to generate (d). (f) Confocal images of Tg(sox10:Eos) animals that were photoconverted at 4 dpf and imaged at 4 and 7 days, demonstrating the Eos photoconversion is stably detected at least 3 days after photoconversion. (g) Quantifications from (f) that demonstrate that photoconversion causes stable labeling of Eos+ cells. (h) Quantification of the abundance of 561-Eos+ microglia in Tg(pu1:Eos) animals that had all microglia photoconverted a 3 dpf and then quantified at 6 dpf. Each data point with connect line represent a single animal. Note the decrease in 561-Eos+ microglia. (i) Quantification of animals depicted in (h) and total number of microglia is quantified. Note that the overall abundance of microglia increases. (j) Quantification of number of microglia in photoconverted (+PC) and non-photconverted (-PC) animals at 6 dpf (p = 0.8665, t test). Scale bar is 10 μm (b, e, f). Descriptive statistics represented in S1 Table. The underlying data can be found in S1 Data. (TIF) [file pbio.3002819.s002.tif]

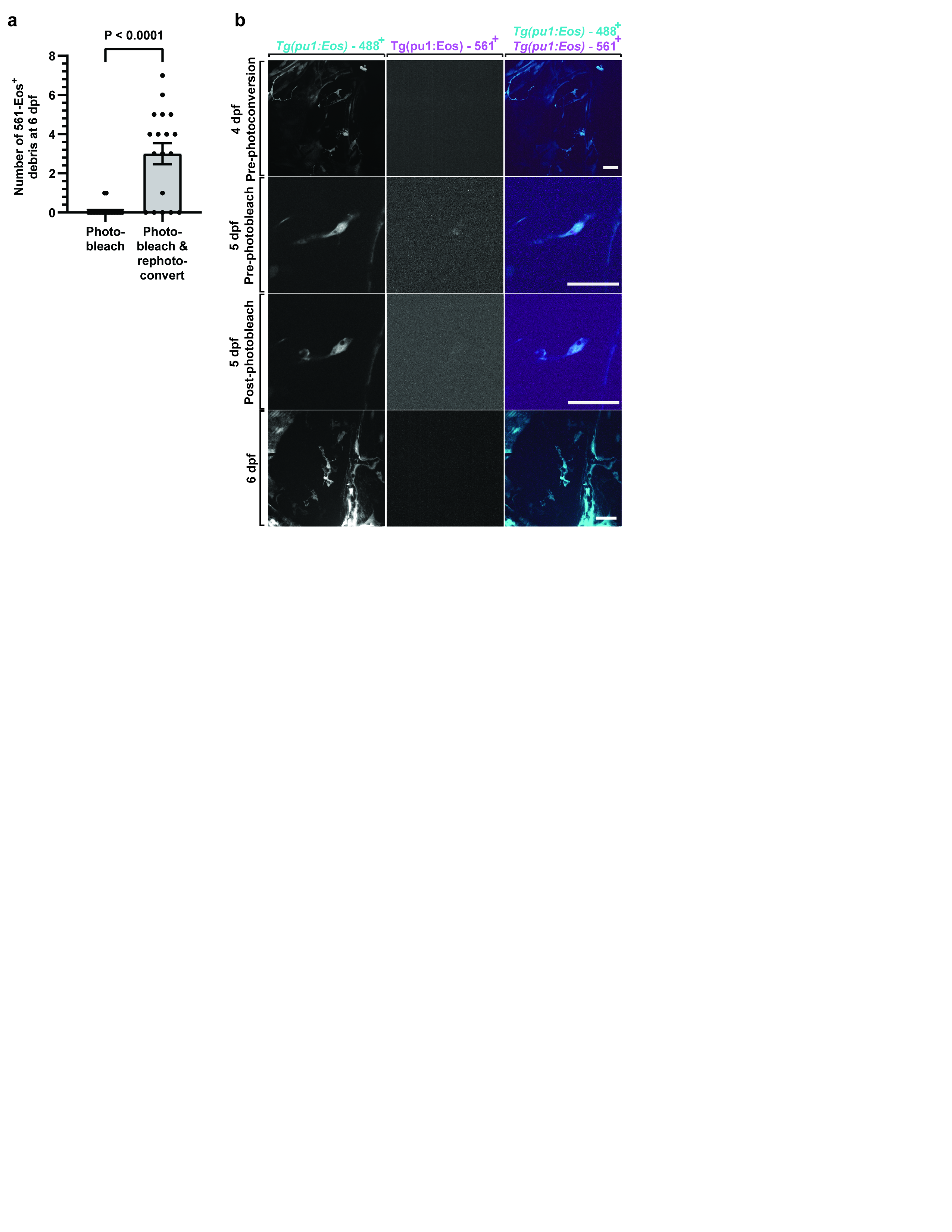

Supplement: S3 Fig — (a) Quantification of the abundance of 561-Eos+ debris in Tg(pu1:Eos) animals that had microglia photoconverted at 4 dpf, and then the debris was photobleached at 5 dpf (photo-bleach) and images were captured at 6 dpf. This is in contrast to the quantification of 561-Eos+ debris from animals that had microglia photoconverted at 4 dpf, photobleached at 5 dpf, rephotoconverted at 5 dpf, and then imaged at 6 dpf (p < 0.0001, t test). (b) Confocal images from the experiment and quantification depicted in (a). Scale bars are 25 μm (b). Descriptive statistics represented in S1 Table. The underlying data can be found in S1 Data. (TIF) [file pbio.3002819.s003.tif]

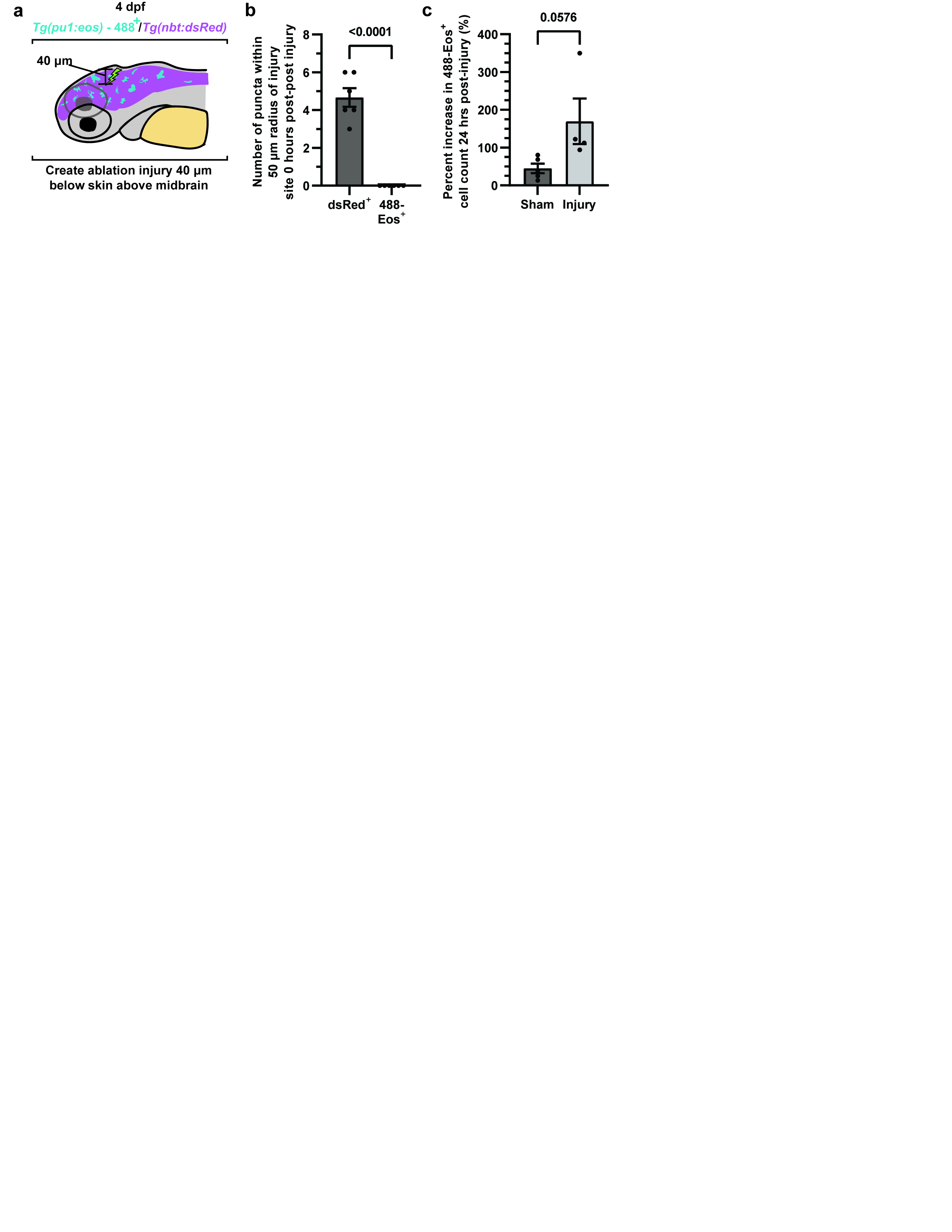

Supplement: S4 Fig — (a) Schematic demonstrating how injuries were created and calibrated in animals. (b) Quantification of the amount of debris immediately after injury at 4 dpf from Tg(nbt:DsRed)+ vs. Tg(pu1:Eos)+ cells in the injury paradigm (p < 0.0001 dsRed+ vs. 488-Eos+, unpaired t test). (c) Quantification of the percentage increase in Tg(pu1:Eos)+ cells in animals 24 h after sham or injury (p = 0.0576 sham vs. injury, unpaired t test). Note the microgliosis consistent with focal brain injuries. Descriptive statistics represented in S1 Table. The underlying data can be found in S1 Data. (TIF) [file pbio.3002819.s004.tif]
